# Pneumococcal carriage in a large Sicilian sample population: impact on the current epidemiological scenario and implications for future vaccination strategies

**DOI:** 10.3389/fcimb.2024.1467320

**Published:** 2024-12-02

**Authors:** Emanuele Amodio, Fabio Tramuto, Valerio De Francisci, Dario Genovese, Valeria Guzzetta, Vincenzo Pisciotta, Arianna Santino, Giulia Randazzo, Giulio Trapani, Giuseppe Vella, Francesco Vitale

**Affiliations:** ^1^ Department of Health Promotion, Mother and Child Care, Internal Medicine and Medical Specialties “P. Giaccone”, University of Palermo, Palermo, Italy; ^2^ Regional Reference Laboratory for Molecular Surveillance of Influenza, Clinical Epidemiology Unit, University Hospital “Paolo Giaccone”, Palermo, Italy

**Keywords:** *Streptococcus pneumoniae*, carriage, colonization, serotypes, epidemiology

## Abstract

**Introduction:**

*Streptococcus pneumoniae* is a prevalent and virulent global pathogen, with colonization being considered a precondition for pneumococcal disease. Understanding colonization is critical for gaining insights into transmission dynamics and developing effective interventions. This study aimed to determine the prevalence of nasopharyngeal colonization and serotype distribution in the Sicilian population.

**Methods:**

Observational study randomly selecting samples belonging to Sicilian individuals whose nasopharyngeal swabs were collected between February 1, 2020, and December 31, 2022. Pneumococcal colonization was determined using PCR for the pneumococcal autolysin (LytA) gene, and positive samples were serotyped.

**Results:**

The study sample consisted of 1,196 individuals, with 17.4% testing positive for the LytA gene. Pneumococcal colonization rates fell from birth to 24 years, with a peak in 0-4-year-olds (aOR=6.9; p<0.001). Colonization was higher in colder months, particularly in December (aOR=2.9, p<0.05) and February (aOR=4, p<0.05). Serotypes 22F and 24ABF exhibited strong colonization and an invasive pneumococcal disease (IPD) risk, whereas serotypes 4, 6AB, 9VA, and 13 had high colonization but a low IPD risk. Serotypes 3 and 8 exhibited considerable IPD risk but low colonization.

**Conclusion:**

Our findings provide insights into pneumococcal colonization mechanisms, influencing serotype prevalence, colonization risk variables, and serotype comparisons for colonization and pathogenicity propensity.

## 1 Introduction

*Streptococcus pneumoniae*, a Gram-positive bacterium classified into more than one hundred immunologically distinct serotypes (Félix et al., 2021), is a highly adapted commensal microbe capable of evading the host’s immune system, leading to a wide spectrum of clinical manifestations, including otitis media, conjunctivitis, sinusitis, and community-acquired pneumonia, with *S. pneumoniae* being the main etiological agent of the latter (Weiser et al., 2018). Furthermore, it may result in more severe conditions such as meningitis, osteomyelitis, and sepsis, collectively referred to as invasive pneumococcal diseases (IPD) (Rudan et al., 2008). Populations at greater risk for pneumococcal infection are children under two years of age and adults aged 65 years or older. Similarly to other respiratory pathogens, the circulation of *S. pneumoniae* is higher during the coldest months. As a result, the incidence of the disease reaches its peak during this period (Gray et al., 1980; Högberg et al., 2007). Seasonality appears to be associated to factors including reduced humidity, indoor crowding, concurrent viral infections, cold temperatures, and air pollution (Domenech de Cellès et al., 2019).

The colonization of the human nasopharynx represents the initial step towards transmission between hosts and the progression from localized to invasive disease. Colonization commonly occurs in infancy and is frequently asymptomatic. Nevertheless, in some individuals, it may lead to infections and, in certain cases, advance to severe clinical outcome (Fleming-Dutra et al., 2014). Several factors, including the virulence of each serotype and the host’s immune response, contribute to the greater likelihood of development from a localized to an invasive form. Risk factors for pneumococcal infection among individuals include influenza coinfection, chronic lung disease, cigarette smoking, and age extremes (Weiser et al., 2018). The global burden of disease attributable to *S. pneumoniae* has markedly decreased thanks to the introduction of multi-valent pneumococcal vaccinations worldwide. Nonetheless, differences exist in the effectiveness of the two main types of anti-pneumococcal vaccines, polysaccharidic (PPSV23) and conjugate (PCV), in preventing the spread of nasopharyngeal carriage (Käyhty et al., 2006). The pneumococcal conjugate vaccine, initially approved for infant use in 2000, has demonstrated efficacy in preventing both IPD and non-invasive forms. It has also been proven effective in reducing nasopharyngeal colonization by the vaccine serotypes (VT). The administration of PCV in children results in concurrent reductions in VT-IPD and carriage among unvaccinated age groups, such as adults. This indicates that pneumococcal carriage in the nasopharynx of infants significantly contributes to the pneumococcal transmission in the community (Fleming-Dutra et al., 2014). Based on the evidence of their effectiveness, pneumococcal conjugate vaccines are currently included in immunization programs in 146 countries (VIEW-hubReport_March2021.pdf, n.d).

Notwithstanding substantial global initiatives, *S. pneumoniae* remains a public health concern. The incidence of IPD in Europe was 14.5 cases per 100,000 children under one year of age (Invasive pneumococcal disease - Annual Epidemiological Report for 2017, 2019), and 187 cases per 100,000 children under five years of age (Wahl et al., 2018). Regrettably, certain regions continue to exhibit inadequate immunization coverage, requiring enhancement (Moreira et al., 2017). Consequently, a significant burden of pneumococcal disease persists, and its associated costs remain elevated (Drijkoningen and Rohde, 2014; Ceyhan et al., 2017). Sicily was the first Italian region to introduce universal mass vaccination against *S. pneumoniae* in its Regional Immunization Plan (RIP) using PCV7 in 2004 and subsequently transitioning to PCV13 in 2010, targeting children under 5 years old with a coverage rate of 95% among newborns. Recently, the 15-valent (PCV15) and 20-valent (PCV20) pneumococcal conjugate vaccines have been developed and authorized for use in the United States and Europe (European Medicines Agency. Prevenar 20 (previously Apexxnar). https://www.ema.europa.eu/en/medicines/human/EPAR/prevenar-20-previously-apexxnar, n.d.; European Medicines Agency. Vaxneuvance (pneumococcal polysaccharide conjugate vaccine (adsorbed). Available at https://www.ema.europa.eu/en/medicines/human/EPAR/vaxneuvance, n.d.).

As of late, the 2023-2025 Italian Vaccination Plan has recommended the administration of PCV+PPSV23 for individuals over 64 years of age and patients at risk aged 5 years old (EpiCentro, n.d).

Recent data from European Surveillance Systems suggest that the epidemiology of pneumococcal disease is undergoing rapid changes at a population level. IPD cases rose from 2014 to 2017. This rise was attributable to serotypes of *S. pneumoniae* not covered by the PCV13 vaccination in subjects over 5 years old (Invasive pneumococcal disease - Annual Epidemiological Report for 2017, 2019; Invasive pneumococcal disease - Annual Epidemiological Report for 2018, 2020). The European Centre for Disease Prevention and Control (ECDC) reports that PCV has conferred herd immunity against pneumococcal infections caused by vaccine serotypes across all age groups. At the same time, the vaccine’s poor ability to cover all serotypes may have facilitated the emergence of new serotypes, a phenomenon known as serotype replacement. Hence, surveillance of circulating serotypes is necessary to evaluate vaccination efforts and guide the development and introduction of updated vaccines. Country-specific factors including pneumococcal disease burden, serotypes distribution, and vaccine cost effectiveness may influence a routine national immunization program. Accordingly, additional information on colonization is required, particularly in regions where the prolonged effects of vaccination may have determined an intense positive pressure on the ecology of this organism. After more than 15 years of vaccine implementation in specific regions, including Sicily, a deeper understanding of the epidemiology of pneumococcal colonization is crucial for elucidating the pathogenesis of pneumococcal disease and assessing its direct and indirect effects, such as herd immunity and replacement.

The primary objective of the present study was to assess nasopharyngeal colonization by *S. pneumoniae*, specifically by measuring colonization rates and serotype distribution in a representative sample of the general population in Sicily.

## 2 Methods

An observational study was conducted to examine *S. pneumoniae* carriage in a sample of the Sicilian general population. To this purpose, a random selection of nasopharyngeal swabs collected between February 1, 2020, and December 31, 2022 at the Sicilian Reference Laboratory of the A.O.U.P. “P. Giaccone” - Palermo (Italy) for the SARS-CoV-2 surveillance was obtained. To this purpose, it is crucial to stress that the study centers around the evaluation and analysis of a particular set of samples gathered during the COVID-19 pandemic to monitor the geographic distribution of SARS-CoV-2 infection in Sicily, Italy. During the study period, more than 50,000 swab samples were available for analysis, including more than 2,000 of them collected from children aged 0 to 5 years.

The randomization process was weighted according to sex, age, expected colonization prevalence in different demographic groups, and the inclusion criteria adopted for the study. The study aimed to have a sample size of 1,152 participants. This number was determined by considering the expected prevalence rate of *S. pneumoniae*, which varies depending on age (ranging from 10% in adults to 30% in the pediatric group), as well as a margin of error (ranging from 3% to 5%) at the 95% level of confidence.

The following characteristics were collected for each subject: sex, date of birth, place of residence, date of biological sampling, manifestation of clinical symptoms (if present), manifestation of clinical signs (if present).

The study was approved by the Ethic Committee of the University Hospital “P. Giaccone” of Palermo - Public Registry Number Protocol n. 10/2021.

### 2.1 Routine testing and genotyping

Nasopharyngeal samples were obtained from each patient and transported to the regional reference laboratory by using standardized flocked swabs which were eluted in Universal Transport Medium (UTM^®^, Copan Diagnostics Inc, USA). To preserve both the quality and integrity of the samples, they have been preserved for a period of maximum six hours after their acquisition and then brought to the laboratory. Once received, the samples were promptly maintained at a temperature of -80°C.

Molecular analyses for *S. pneumoniae* serotypization were conducted in accordance with CDC current protocols (U.S. Centers for Disease Control and Prevention, 2024b, a), partially adapting the methodology reported by Dr. Velusamy (Velusamy et al., 2020) to meet our specific study requirements and objectives.

*Pneumococcus* DNA was extracted and purified using the QIAamp DNA mini kit (Qiagen, Valencia, CA, USA) following the manufacturer’s protocol with a few modifications. Briefly, 180 μL of each sample was added with 80 μL of lysozyme (20 mg/mL in 20 mM Tris-HCl, pH 8.0, 2 mM EDTA, 1.2% Triton) and incubated at 37°C for 30 min. Following the addition of 20 μL of proteinase K (0.5 mg/mL in Tris-HCl 20 mM, pH 8.0) and 200 μL of the lysis buffer provided with the kit, the mixture was further incubated at 56°C per 30 min, and the enzyme subsequently denatured at 95°C for 15 min. All other steps were followed as per the manufacturer’s instructions to a final volume of 100 μL elution buffer. Carriage of *S. pneumoniae* was evaluated by means of a single-plex real-time PCR (rt-PCR) assay for the detection of pneumococcal autolysin (*LytA*) gene sequence, and a sample was assumed to be negative if there was no increase in fluorescent signal after 40 rt-PCR cycles. All *LytA* positive samples were included in serotyping analysis by means of a set of 40 different real-time assays arranged in 12 triplex and 2 duplex, respectively, for the detection of specific genetic segments of pneumococcal serotypes/serogroups covered by vaccine formulation in use or next to be used in Sicily (PPV23, PCV13, PCV15, and PCV20). Nevertheless, the total number of examined serotypes/serogroups was lower because of the redundance of some tests, such as 6ABCD, 6AB, 6CD, 22AF, 22A and 22F, resulting in the analysis of 37 serotypes/serogroups (1, 2, 3, 4, 5, 6AB, 6CD, 7AF, 7CB2, 8, 9LN, 9VA, 10A, 11ADE, 12F44, 13, 14, 15BC, 16F, 17F, 18ABCF, 19A, 19F, 20, 21, 22A, 22F, 23A, 23B, 23F, 24ABF, 31, 33AF37, 34,35A, 35B, 38). To this end, all molecular tests were carried out in duplicate, including both negative and plasmid positive controls of each serotype.

Real-time RT-PCR was performed to assess for the presence of SARS-CoV-2 (N=1,196) in all samples. RNA of viral pathogens was extracted using QIAamp Viral RNA extraction kit (QIAGEN) according to the manufacturer’s suggested protocol and the RNA eluted from the spin column in 60 μL of elution buffer. Eluted RNA was divided into aliquots and stored immediately at -80°C until further use.

An External Quality Assessment for molecular diagnostics was conducted utilizing Quality Control for Molecular Diagnostics (QCMD, https://www.qcmd.org/en/) samples to evaluate the clinical accuracy of our laboratory, including assays targeting *Streptococcus pneumoniae*.

The specificity of our protocols is periodically verified on pneumococcal isolates collected from patients with invasive pneumococcal disease, which routinely undergo whole genome sequencing to further confirm the etiology, and each serotype involved in the clinical condition.

### 2.2 Statistical analyses

The epidemiological and laboratory data were recorded in an electronic database and analyzed to calculate descriptive statistics, such as carriage rates and serotype distribution, described as proportion of all the isolates, including both vaccine and non-vaccine serotypes. Additionally, the possible association between pneumococcal colonization and socio-demographic characteristics was evaluated. The chi-square test (or Fisher’s exact test when appropriate) was applied to compare colonization prevalence among different groups. Pneumococcal serotypes were classified as having a high colonization prevalence or a high IPD risk if they were over the 80^th^ percentile of the relevant distribution.

Adjusted-odds ratios (aOR) and 95% confidence intervals (CI) were calculated by a multivariable logistic regression analysis evaluating the factors that may modify the risk of pneumococcal colonization. A p-value equal to or less than 0.05 was considered statistically significant. All the statistical analyses were performed with R statistical software version 4.2.2, and the figures were designed by using Microsoft Excel.

## 3 Results

As reported in **Table 1**, the study population included a total of 1,196 subjects including 657 (55.0%) females and 539 (45.0%) males. About a half of the study subjects was 0 to 14 years old (46.2%) and almost all participants were recruited in 2020 (n=569, 47.6%) and in 2021 (n=536; 44.8%). A large majority of the sample did not present any clinical manifestation (n=1,134; 94.8%) at the time of biological sampling, whereas 62 (5.2%) subjects reported some symptoms.

**Table 1:** General characteristics of the population sample recruited in the study (n=1,196 subjects).

	N, (%)
**Total sample**	1,196 (100%)
Sex	
• Females	657 (55.0%)
• Males	539 (45.0%)
Age group	
• - 0 to 14 yrs old	553 (46.2%)
• - >14 yrs old	643 (53.8%)
Age	
• 0 to 4 yrs old	172 (14.4%)
• 5 to 14 yrs old	381 (31.8%)
• 15 to 24 yrs old	42 (3.5%)
• 25 to 44 yrs old	197 (16.5%)
• 45 to 64 yrs old	225 (18.8%)
• 65 to 74 yrs old	84 (7.0%)
• ≥75 yrs old	95 (7.9%)
Year of sampling	
• 2020	569 (47.6%)
• 2021	536 (44.8%)
• 2022	91 (7.6%)
Clinical presentation at the sampling time	
• No symptoms	1,134 (94.8%)
• Mild symptoms	62 (5.2%)
• Severe symptoms	0 (0%)

**Figure 1:**
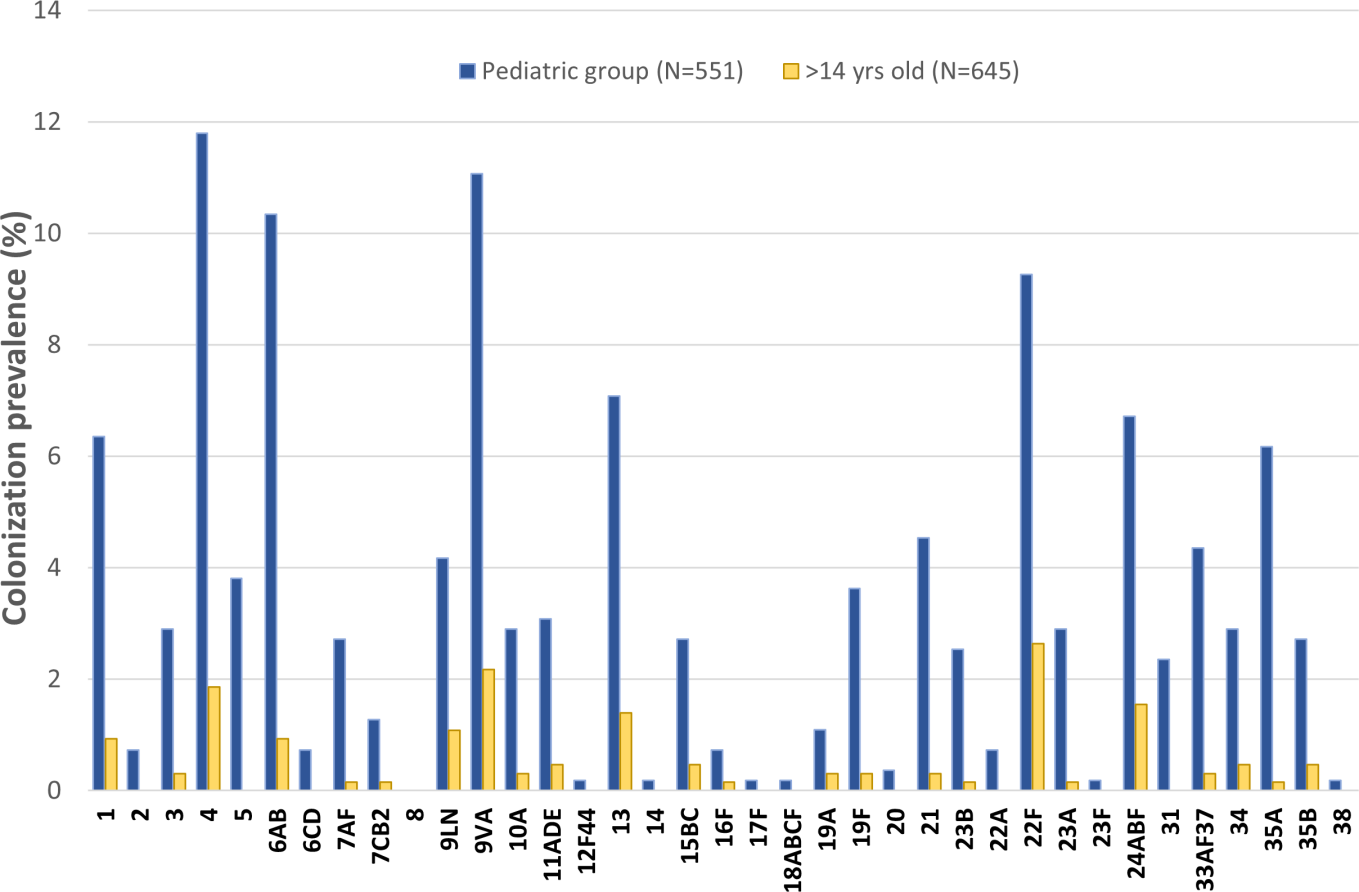
Prevalence of pneumococcal colonization according to the different serotypes in pediatric (N=551 subjects) and over 14 years old (N=645 subjects) populations (in bold PCV13 serotypes).

**Figure 1** illustrates the prevalence of pneumococcal colonization in both pediatric and over 14 years old populations, according to the different serotypes/serogroups. The figure also depicts the prevalence of VT or non-VT. The most often isolated serotypes/serogroups in the pediatric group were: 4 (11.8%), 9VA (11.1%), 6AB (10.3%), and serotype 22F (9.3%). In the group of individuals aged over 14 years and older, the most frequently represented serotypes/serogroups were: 22F (2.6%), 9VA (2.2%) and 4 (1.9%). The PCV13 vaccine targets three out of the four serotypes/serogroups that have the highest prevalence of colonization (4, 6AB and 9VA).

The total number of serotypes carried per each person was also investigated. Among individuals aged 0-14 years, 4% harbored 1 serotype and 3% harbored 7 or more serotypes. In the over 14 years old group, 1 serotype was detected in 1% of the subjects, whereas 3% of participants exhibited the contemporary presence of 3 or more serotypes.

As reported in **Table 2**, 17.4% of recruited subjects resulted positive to *LytA* gene. The prevalence of pneumococcal colonization had a decreasing trend from 0 to 24 years, with a peak in the 0 to 4 years group (aOR 6.9; p<0.001). In the adult group, the prevalence of colonization has a downward trend, with a peak in the 25-44 years group. Pneumococcal colonization rate was also evaluated in relationship with the month in which biological sample was performed. Colder months as December, and February were characterized by higher colonization rates (aOR=2.9, p<0.05; aOR=4, p<0.05) with respect to other months.

**Table 2:** Prevalence of pneumococcal colonization according to the different investigated characteristics.

	LytA positive subjects N/Tot (%)	p-value	aOR (95% CI)
**Total**	208/1,196 (17.4%)	–	–
Sex			
• M	100/539 (18.6%)	0.34	–
• F	108/657 (16.4%)		
Age group			
• 0 to 4 yrs old	76/172 (44.2%)	<0.001	6.9 (3.9-12.2)^c^
• 5 to 14 yrs old	93/381 (24.4%)		3.0 (1.7-5.1)^c^
• 15 to 24 yrs old	1/42 (2.4%)		0.24 (0.03-1.9)
• 25 to 44 yrs old	19/197 (9.6%)		*Referent*
• 45 to 64 yrs old	15/225 (6.7%)		0.7 (0.3-1.4)
• 65 to 74 yrs old	2/84 (2.4%)		0.2 (0.1-1.1)
• ≥ 75 yrs old	2/95 (2.1%)		0.19 (0.1-0.8)^a^
Year of sampling			
• 2020	107/569 (18.8%)	0.47	
• 2021	86/536 (16.0%)		–
• 2022	15/91 (16.5%)		
Month of sampling			
• January	27/103 (26.1%)	<0.001	2.4 (0.9-6.4)
• February	31/104 (29.8%)		3.0 (1.2-7.6)^a^
• March	21/142 (14.8%)		1.2 (0.4-3.0)
• April	20/191 (10.47%)		0.9 (0.3-2.3)
• May	15/103 (14.6%)		1.3 (0.5-3.6)
• June	7/80 (8.7%)		*Referent*
• July	8/66 (12.1%)		1.6 (0.5-5.1)
• August	3/36 (8.3%)		1.6 (0.4-7.0)
• September	12/87 (13.8%)		1.6 (0.5-4.4)
• October	12/76 (15.8%)		1.2 (0.4-3.6)
• November	28/124 (22.6%)		1.2 (0.4-3.6)
• December	24/84 (28.6%)		2.9 (1.1-7.4)^a^

Letters in superscript refer to p-values. In detail: ^a^<0.05; ^b^<0.01; ^c^<0.001.

**Figure 2:**
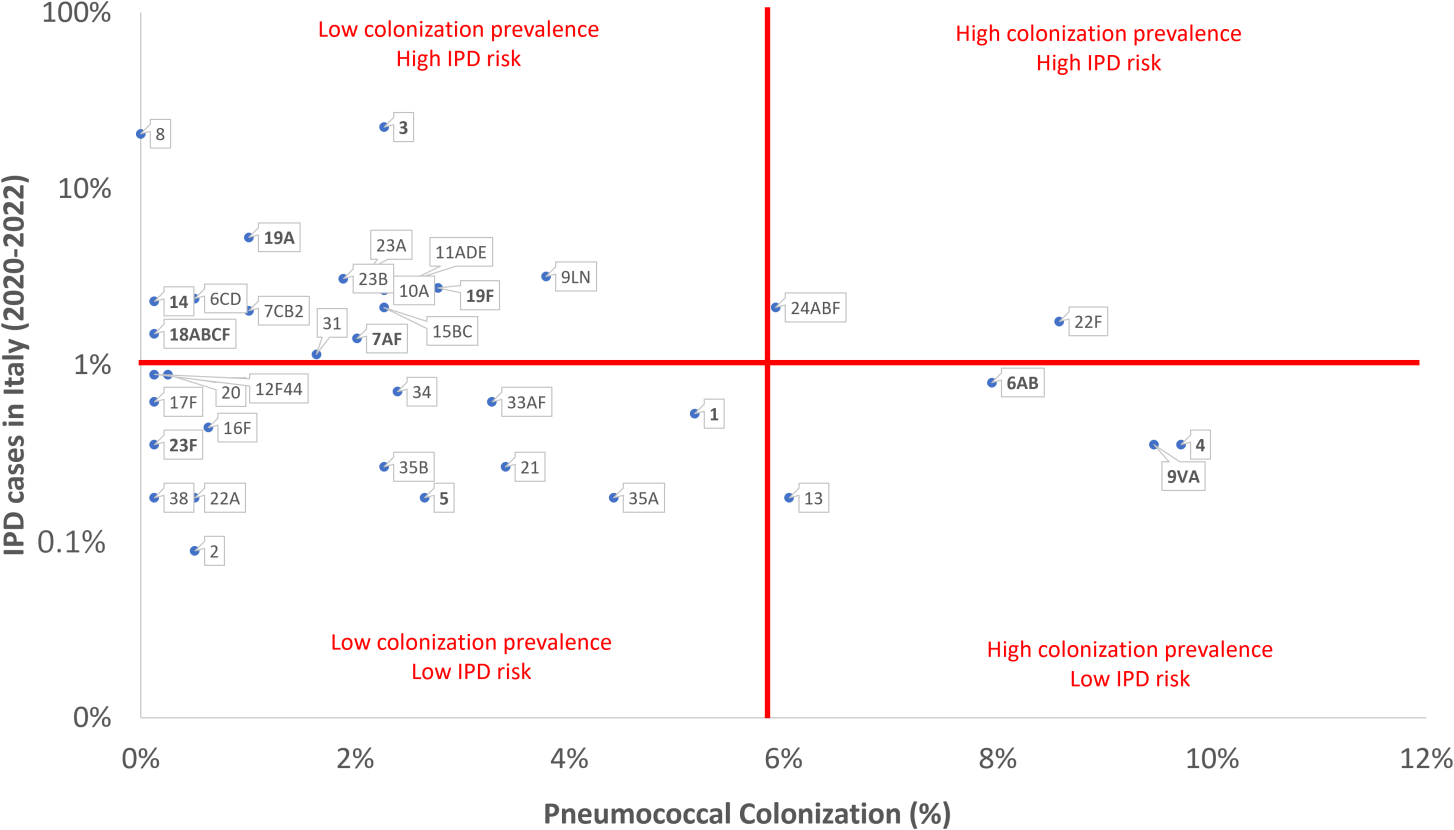
Comparison between the prevalence of pneumococcal serotypes/serogroups reported in the invasive pneumococcal disease (IPD) Italian surveillance system and the pneumococcal serotypes/serogroups colonization frequency observed in the present study (in bold PCV13 serotypes).

**Figure 2** reports a comparison between the prevalence of pneumococcal serotypes isolated from the invasive pneumococcal disease through the Italian surveillance system (Rapporti ISS, n.d) and the pneumococcal serotype colonization frequency observed in the present study. We observed two serotypes with both high colonization prevalence and a high IPD risk (22F and 24ABF). Four serotypes had high colonization prevalence but a low IPD risk (4, 6AB, 9VA and 13). Pneumococcal serotypes 3 and 8 showed a very high IPD risk but low pneumococcal colonization prevalence.

Finally, we observed that subjects colonized with *Pneumococcus* had a lower risk of concurrent SARS-CoV-2 infection (10.6% *vs*. 17.5% in non-colonized subjects, p<0.01).

## 4 Discussion

*Streptococcus pneumoniae* is undeniably one of the most common and virulent pathogens worldwide, with colonization being a precondition for the onset of pneumococcal disease (Jochems et al., 2017). Understanding the role of pneumococcal colonization is essential for gaining valuable insights into the dynamics of pneumococcal transmission and devising more effective measures to combat it.

Our study highlights that pneumococcal colonization is a very common event, especially in the pediatric population, affecting around one third of subjects. In contrast, a lower prevalence of pneumococcal colonization can be observed among older subjects. The results of our study closely align with those reported by international literature. Pneumococcal colonization exhibits a distinct age-dependent trend, with a higher prevalence in children compared to adolescents and adults (Althouse et al., 2017; Chamorro et al., 2023). Several studies reported that colonization rates among infants ranges from 20% to 60%, with populations with limited access to healthcare exhibiting higher rates (Brooks and Mias, 2018). Several biological factors, including immature immune system, close contact to peers in childcare settings, lack of pre-existing immunity, and social behaviors such as sharing toys, food, and beverages, can contribute to the elevated incidence of colonization in children. While several studies have examined pneumococcal colonization in infants, less is known about colonization in older adults, particularly in low- and middle-income countries (Neal et al., 2022). Nonetheless, research suggests that colonization rates in older adults are generally lower compared to children (Tramuto et al., 2017; Brooks and Mias, 2018).

Alongside age, several other major factors could contribute to explaining higher colonization rates. Our findings suggest that pneumococcal colonization had a higher prevalence in the colder months, namely December, January, and February (29%, 26% and 30%, respectively) compared to other months. As stated by the research group led by Domenech de Cellès, our knowledge of pneumococcal seasonality remains limited (Domenech de Cellès et al., 2019). However, evidence suggests that a variety of factors, including environmental conditions such as temperature and humidity (Althouse et al., 2017; Domenech de Cellès et al., 2019), as well as social dynamics like school holidays that foster contacts between adults and children, may contribute to the winter peak of colonization. This increased contact serves as one of the main transmission routes for pneumococcal infection in adults, constituting a risk factor (Brooks and Mias, 2018). However, there are also several additional factors that might account for this peak of colonization during the winter season, such as dry indoor air from heating, weakened immune response, and concurrent viral infections due to influenza, hRSV, and SARS-CoV-2. Regarding the increased host susceptibility during the colder months, a recent study revealed significant seasonal fluctuations in multiple markers of human immunity, namely the presence of a marked pro-inflammatory response, which is recognized to strengthen the persistence of *S. pneumoniae* in the nasopharynx, during the winter season (Dopico et al., 2015; Domenech de Cellès et al., 2019). A prospective cohort study in England found that individuals with a concomitant SARS-CoV-2 infection had a lower prevalence of pneumococcal colonization. Out of 160,886 laboratory-confirmed cases of SARS-CoV-2 infections, only 88 cases had both pneumococcal and SARS-CoV-2 coinfection (Amin-Chowdhury et al., 2021).

As second point of our study, we observed a significant colonization rate associated with serotypes/serogroups 4, 6AB, 9VA (included in PCV13), and 22F (not included in PCV13, but included in both PCV15 and PCV20 formulations). These serotypes accounted for about one third of all identified serotypes. Additional assessment is required for the findings concerning serotype 4, one of the vaccine serotypes since the introduction of PCV7 particularly prevalent in the studied sample of this study. A 2018 systematic review with meta-analysis investigates the temporal patterns of different serotypes in the etiology of IPD following the introduction of the PCV13 vaccine (Balsells et al., 2018). The review, focusing on individuals under 2 years of age, concludes that several non-vaccine serotypes had a lower risk of causing invasive disease compared to 19A (odds ratio 0.1-0.3) (Balsells et al., 2018). Among the serotypes examined, serotype 4 is nearly absent, mentioned only in the Supplementary File of the cited article. It can be deduced that the invasiveness of serotype 4 has declined over time after the implementation of vaccine formulations commonly used in the pediatric population. However, the recent work by Pérez-García et al. elucidates the ongoing evolution of the IPD trend (Pérez-García et al., 2024). This study examines IPD surveillance data in Spain from 2019 to 2023, revealing that the majority of IPD cases attributed to serotype 4 occur in young adults, with a significant rise throughout the specified time frame (Pérez-García et al., 2024). Additionally, it is reasonable to state that the incidence of serotype 4-dependent IPD is limited, with only 3 cases reported in 2023 among those aged under 2. The data presented in this study may provide evidence that the invasiveness of serotype 4 has considerably decreased, particularly among children under the age of 4, as a result of the vaccine efficacy in reducing the chance of hospitalization in the case of *S. pneumoniae* serotype 4 infection.

The prevalence of specific serotypes may vary based on geographical region and time, influenced by vaccination programs, antibiotic usage, and population dynamics. Surveillance studies play a vital role in continuous monitoring of pneumococcal serotypes distribution and in guiding vaccination strategies. Nevertheless, we firmly believe that further information could be obtained by comparing the prevalence of pneumococcal serotypes isolated from the IPDs notified through the Italian surveillance system (Rapporti ISS, n.d) and the frequency of pneumococcal serotype colonization observed in the present study. We discovered that two serotypes, 22F and 24ABF, exhibited both a high colonization prevalence and a high IPD risk. Alternatively, pneumococcal serotype 8 showed a significant IPD risk but had a low colonization prevalence, suggesting a possible higher virulence. To the best of our knowledge, this study is one of the first efforts to compare pneumococcal colonization and disease rates in Sicily. However, it is important to acknowledge that other researchers have examined the invasiveness of circulating *S. pneumoniae* serotypes across different geographical regions, including the UK, Spain, and US, revealing distinctive distribution patterns of serotype prevalence and varying levels of invasiveness, particularly regarding serotypes 1, 8, and 12F (Bennett et al., 2021; Bertran et al., 2024; Losada-Castillo et al., 2024). Therefore, further studies should be conducted to comprehend the complex interplay of bacterial, host, and environmental factors that may contribute to the progression from colonization to disease. The presence of a polysaccharide capsule surrounding pneumococcal bacteria may play a crucial role in determining whether the bacterium colonization evolves into disease or not. Animal models have demonstrated that certain serotypes have capsules that are less likely to provoke an immune response or are less effective at evading the immune system. Consequently, these serotypes are more prone to cause asymptomatic colonization instead of disease (Weiser et al., 2018).

Some results should be interpreted cautiously due to potential confounding factors and the limitations of the study design. This retrospective study analyzes a subset of swabs collected for regional SARS-CoV-2 surveillance, highlighting that the vial type, transport medium, and broth collecting procedure may not conform to the recommendations for all age groups investigated. Nevertheless, it is important to highlight that while trans-nasal sampling is widely recognized as the standard method for the pediatric population, its application in adults is still a subject of controversy when compared to trans-oral sampling procedure (Miellet et al., 2023). Another limitation that should be acknowledged is that our study sample may not fully representative of the Italian population from which the IPD cases were obtained. Furthermore, our study does not consider some factors such as smoking habits or environmental factors that could change both the risk of colonization and the specific pneumococcal serotype responsible. Moreover, regarding the statistical methods and data correlations evaluated, non-conventional incidence measures have been adopted. Indeed, despite the case-carrier ratio (CCR) could have been a suitable statistical measure for the purposes of this study (Vissers et al., 2018), it is important to note that CCR, as a ratio, generally does not possess the capacity to effectively communicate information regarding IPD prevalence and colonization rates. Consequently, recognizing the significance of preserving information regarding both variables, the research group opted to create a custom graph that enables the reader to access data on each pneumococcal serotype. Finally, it is essential to be aware of the difficulties of obtaining a clear and unambiguous response concerning the specificity of serotype assays, as evidenced by prior research (Almeida et al., 2021; Miellet et al., 2022). Consequently, the findings related to the prevalence of specific serotypes and serogroups, especially serotype 4 and serogroup 9, require critical and cautious evaluation, with the goal that this issue will be explored more comprehensively in future research.

Despite of these potential limitations, we are confident that the previously reported findings could significantly contribute to our knowledge of the complex phenomena involved in pneumococcal colonization and the correlation between colonization and disease.

## Data Availability

The datasets presented in this article are not readily available because they contain sensitive data, protected by the current Italian laws on privacy and sensitive data protection. Requests to access the datasets should be directed to the Corresponding Author.
